# Overcoming resistance to antibody-drug conjugates: mechanisms and emerging strategies

**DOI:** 10.1093/oncolo/oyag020

**Published:** 2026-02-03

**Authors:** Aki Inase, Shiro Kimbara, Eli Imamura, Mitch A Phelps, Hironobu Minami

**Affiliations:** Division of Medical Oncology and Hematology, Department of Medicine, Kobe University Graduate School of Medicine, Kobe, 650-0017, Japan; Division of Pharmaceutics and Pharmacology, College of Pharmacy, The Ohio State University, Columbus, OH, 43210, United States; Division of Medical Oncology and Hematology, Department of Medicine, Kobe University Graduate School of Medicine, Kobe, 650-0017, Japan; Timele Clinic, Tokyo, 167-0022, Japan; Division of Pharmaceutics and Pharmacology, College of Pharmacy, The Ohio State University, Columbus, OH, 43210, United States; Pelotonia Institute for Immuno-Oncology, OSUCCC - James, The Ohio State University, Columbus, OH, 43210, United States; The James Comprehensive Cancer Center, The Ohio State University, Columbus, OH, 43210, United States; Division of Medical Oncology and Hematology, Department of Medicine, Kobe University Graduate School of Medicine, Kobe, 650-0017, Japan; Cancer Center, Kobe University Hospital, Kobe, 650-0017, Japan

**Keywords:** antibody–drug conjugates, ADC resistance, resistance mechanisms, combination therapy, next-generation ADCs, preclinical studies

## Abstract

**Background:**

Antibody-drug conjugates (ADCs) combine a tumor antigen-specific monoclonal antibody with a cytotoxic payload via a linker, enabling selective delivery of cytotoxic agents to cancer cells while minimizing damage to healthy tissues. This approach has revolutionized cancer treatment; however, despite these advantages, resistance to ADCs has emerged as a significant barrier to long-term efficacy.

**Methods:**

In this review, we summarize and analyze molecular pathways implicated in ADC resistance across multiple cancer types, including triple-negative breast cancer, non-small cell lung cancer, pancreatic cancer, and relapsed/refractory acute myeloid leukemia. We further examine emerging strategies to overcome resistance, with particular emphasis on combination therapies and the development of next-generation ADCs.

**Results:**

Accumulating evidence indicates that identifying and targeting key resistance-associated pathways can expand the population of patients who benefit from ADC therapy and extend the therapeutic lifespan of these agents. We highlight representative preclinical studies that have elucidated resistance mechanisms and demonstrate potential approaches to restore or enhance ADC efficacy.

**Conclusion:**

Understanding and overcoming ADC resistance is essential as these agents continue to expand into new therapeutic settings. By bridging basic mechanistic insights with translational and preclinical evidence, this review provides a comprehensive framework for addressing ADC resistance and informs future strategies for optimizing cancer treatment.

Implications for Practice Resistance to antibody–drug conjugates (ADCs) represents a major limitation to their long-term clinical efficacy. By summarizing key resistance mechanisms and emerging strategies, including combination approaches and next-generation ADC design, this review provides a framework to inform the anticipated clinical course of treatment among current ADC therapies, rational therapeutic development, and the design of next-generation ADCs aimed at improving durability of response.

## Introduction

Antibody-drug conjugates (ADCs) have emerged as a transformative class of cancer therapy, combining the specificity of monoclonal antibodies (mAbs) with the potent cytotoxicity of small molecule drugs (payloads). Over the past decade, advances in ADC design have significantly improved therapeutic efficacy while addressing challenges related to resistance and toxicity. Despite these advances, resistance mechanisms remain a major obstacle, limiting the long-term success of ADC therapy. Resistance can arise from a variety of factors, including loss of antigens, altered intracellular trafficking, and upregulation of drug efflux transporters. To overcome these limitations, researchers are developing novel payloads and linkers, bi-specific and multi-specific ADCs, and personalized treatment strategies. In this review, we discuss the latest innovations in ADC technologies, mechanisms of resistance observed in clinical trials, and strategies to enhance the efficacy of ADC-based therapies.

## Structure of ADCs and their mechanism of action

**Figure 1:**
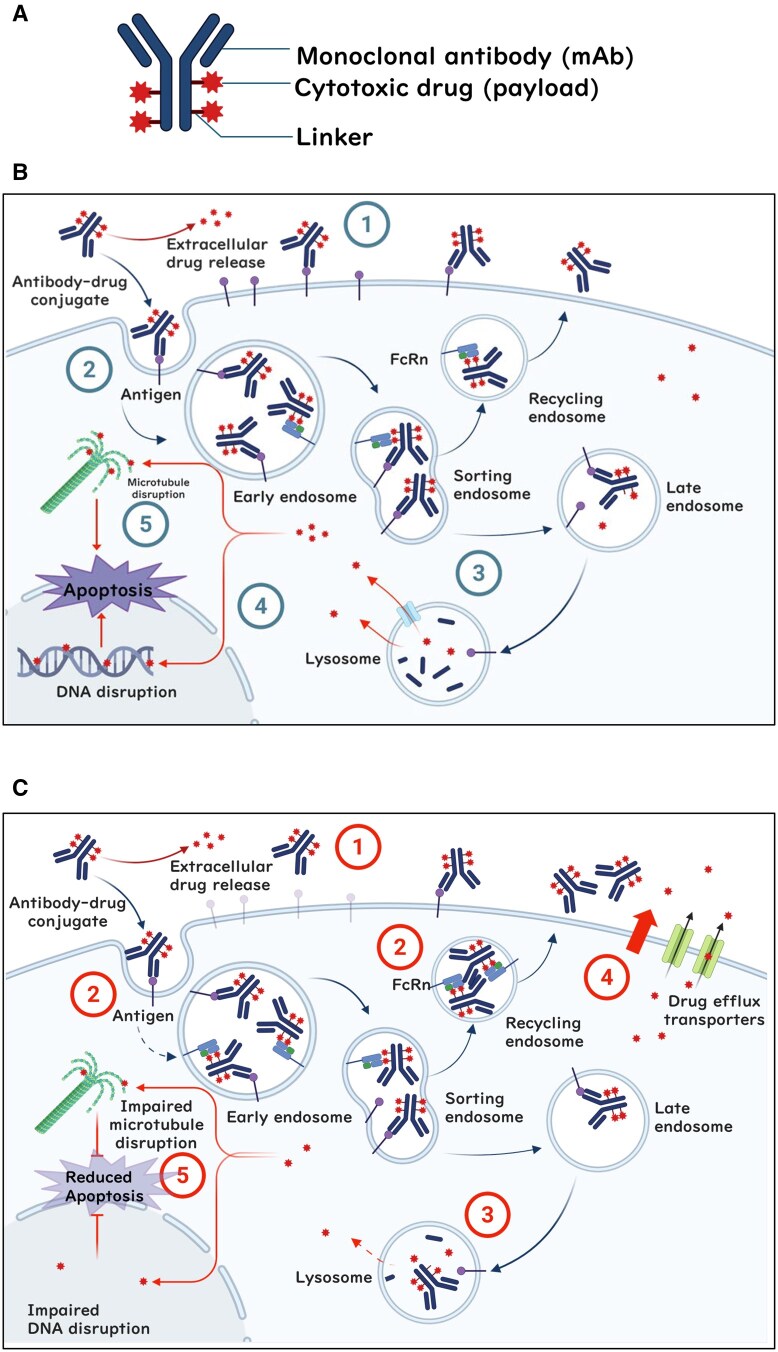
Structure and mechanisms of antibody-drug conjugate (ADC) activity and resistance. (A) ADCs consist of a monoclonal antibody (mAb) that binds to a specific antigen on cancer cells, a linker that connects the antibody to a cytotoxic payload, and a potent chemotherapy drug responsible for killing cancer cells. (B) The cytotoxic effect of ADCs involves multiple steps. First, the ADC binds to its target antigen and forms an ADC-antigen complex ①. This complex is then internalized into the cancer cell via receptor-mediated endocytosis ② and via endosomal trafficking is either recycled via neonatal Fc receptor (FcRn) binding or is transferred to the lysosomal compartment, where it undergoes degradation ③. The released cytotoxic payload induces cell death by either causing DNA damage ④ or disrupting microtubule stability, leading to apoptosis ⑤. (C) The major mechanisms contributing to ADC resistance include antigen downregulation or loss (primary/acquired): Both low baseline antigen expression and treatment-induced antigen reduction limit ADC binding and payload delivery ①. Reduced antigen internalization or altered intracellular trafficking (primary): Some tumors intrinsically show inefficient internalization or preferential routing through non-productive pathways (e.g., caveolae-mediated uptake or FcRn-mediated recycling), reducing lysosomal delivery of ADCs ②. Lysosomal dysfunction and impaired payload release (acquired): Treatment pressure can cause lysosomal alkalinization, reduced protease activity, or loss of transporters such as SLC46A3, leading to ineffective payload liberation despite ADC uptake ③. Increased expression of drug efflux pumps (primary/acquired): High baseline or treatment-induced expression of drug efflux transporters (e.g., MDR1, ABCG2) reduces intracellular accumulation of released payloads by actively pumping them out of the cell ④. Dysregulation of apoptotic pathways (primary/acquired): Elevated levels or treatment-induced upregulation of anti-apoptotic proteins (e.g., BCL-2, BCL-XL, MCL-1) diminish the cytotoxic effects of ADC payloads, even when internalization and release occur normally ⑤. Figure was created with BioRender.com.

ADCs are a targeted cancer therapy comprising three components, the targeting mAb, the cytotoxic payload, and the linker that couples those two components together (Figure 1A).^1^ The rationale for ADCs is to deliver a potent anticancer drug directly to tumor cells while sparing healthy cells, thereby increasing efficacy and minimizing side effects.^2^ ADCs can efficiently destroy targeted cancer cells by performing four main steps, the first three of which are effectively driven by complex combination of targeting antibody and payload pharmacokinetics^3^ (Figure 1B): First, the ADC circulates systemically until it binds to its specific antigen on the surface of a cancer cell. Second, the ADC is most often internalized by the cancer cell through receptor-mediated endocytosis. Third, the ADC enters the endosomal-lysosomal pathway,^4^ where the cytotoxic payload is released. Fourth, the released drug typically exerts its effect by interfering with vital cell processes (DNA synthesis, microtubule function, etc.), leading to cell death.^2^ These four steps underly resistance mechanisms in ADC therapy.^5^ Because binding to tumor cell antigens is the first step in the mechanism of action of ADCs, antigen expression levels usually influence therapeutic efficacy.^6–10^ However, expression levels vary within tumors and among patients, and antigen expression does not always correlate with therapeutic outcomes.^11^^,^^12^ Additionally, clonal evolution in antigen-negative tumor cells may contribute to development of acquired resistance.^13^ Similarly, efficiency of uptake into cancer cells, linker cleavage and payload release, and sensitivity of cells to the released payload all impact therapeutic efficacy. For example, impaired endocytosis and reduced lysosomal function may reduce activation of ADCs, resulting in insufficient induction of cell death.^14^ Importantly, efficiency of drug release also depends on the type of linker used. Drug release typically relies on enzymatic cleavage or acidic conditions within the lysosome for cleavable linkers, whereas non-cleavable linkers require complete degradation of the antibody component within the lysosome to release the active drug. Changes in these multiple steps are contributors to primary resistance and acquired resistance after treatment.

## FDA-approved ADCs

ADCs have been approved by the United States Food and Drug Administration (FDA) for treating various types of cancer, either as monotherapy or in combination with other agents.^15^^,^^16^ Each ADC targets a specific antigen and uses a unique payload and linker combination. At present, there are 14 ADCs approved by the FDA, with over 200 additional ADCs at various stages of clinical trials. Table 1 lists FDA-approved ADCs, as of December 2025. The payload defines the cytotoxic activity of ADCs, with three main categories used in those that are FDA-approved: DNA-damaging agents (calicheamicin derivative [ozogamicin], pyrrolobenzodiazepine dimer [SG3199]), microtubule inhibitors (maytansinoids: emtansine [DM1], ravtansine [DM4]; and monomethyl auristatin E [MMAE]), and topoisomerase I inhibitors (deruxtecan [DXd], and SN-38). The linker plays a crucial role determining when and where the payload is released. Cleavable linkers are sensitive to pH (acid-cleavable) or enzymatic processing (enzyme-cleavable) and facilitate payload release within the endosomal-lysosomal compartments, where these conditions are prevalent. With non-cleavable linkers, payload activation of ADCs such as trastuzumab emtansine (T-DM1) requires complete degradation of the ADC in lysosomes, which confers superior systemic stability. Optimizing the payload-linker combination is critical for balancing efficacy and safety in ADC development.

**Table 1.** Food and Drug Administration (FDA)-approved antibody-drug conjugates (ADCs).

ADC	Common name	Target	mAb	Linker	Payload/active agent	Payload action	DAR	Site	FDA-approved indications	First FDA approved
**Mylotarg**	Gemtuzumab Ozogamicin (GO)	CD33	IgG4	Acid cleavable	Ozogamicin/calicheamicin	DNA cleavage	2-3	Lys (random)	CD33+ AML	May 17, 2000^*1^
**Adcetris**	Brentuximab Vedotin (BV)	CD30	IgG1	Enzyme cleavable	MMAE/auristatin	Microtubule inhibitor	4	Cys (random)	cHL, sALCL, PTCL	Aug 19, 2011
**Kadcyla**	Trastuzumab Emtansine (T-DM1)	HER2	IgG1	Non-cleavable	DM1/maytansinoid	Microtubule inhibitor	3.5	Lys (random)	HER2+ BC	Feb 22, 2013
**Besponsa**	Inotuzumab Ozogamicin (IO)	CD22	IgG4	Acid cleavable	Ozogamicin/calicheamicin	DNA cleavage	6	Lys (random)	B-ALL	Aug 17, 2017
**Polivy**	Polatuzumab Vedotin (PV)	CD79b	IgG1	Enzyme cleavable	MMAE/auristatin	Microtubule inhibitor	3.5	Cys (random)	DLBCL	Jun 10, 2019
**Padcev**	Enfortumab Vedotin (EV)	Nectin4	IgG1	Enzyme cleavable	MMAE/auristatin	Microtubule inhibitor	3.8	Cys (random)	UC	Dec 18, 2019
**Enhertu**	Trastuzumab Deruxtecan (T-Dxd)	HER2	IgG1	Enzyme cleavable	DXd/camptothecin	TOP1 inhibitor	8	Cys (random)	HER2+ BC, HER2-low BC, HER2+ GC, HER2-mut NSCLC, HER2-IHC3+ solid tumors	Dec 20, 2019
**Trodelvy**	Sacituzumab Govitecan (SG)	TROP2	IgG1	Acid cleavable	SN-38/camptothecin	TOP1 inhibitor	7.6	Cys (random)	mTNBC, HR+/HER2- BC, UC	Apr 22, 2020
**Blenrep**	Belantamab mafodotin (Belamaf)	BCMA	IgG1	Non-cleavable	MMAF/auristatin	Microtubule inhibitor	4	Cys (random)	MM	Aug 5, 2020^*2^
**Zynlonta**	Loncastuximab Tesirine (Lonca)	CD19	IgG1	Enzyme cleavable	SG3199/PBD dimer	DNA cleavage	2.3	Cys (random)	DLBCL	Apr 23, 2021
**Tivdak**	Tisotumab Vedotin (TV)	Tissue Factor	IgG1	Enzyme cleavable	MMAE/auristatin	Microtubule inhibitor	4	Cys (random)	CC	Sep 20, 2021
**Elahere**	Mirvetuximab Soravtansine	FRα	IgG1	Cleavable disufide linker	DM4/maytansinoid	Microtubule inhibitor	3.5	Lys (random)	FRα+ OvCa	Nov 14, 2022
**Datroway**	Datopotamab Deruxtecan (Dato-Dxd)	TROP2	IgG1	Tetrapeptide-based cleavable linker	DXd/camptothecin	Topoisomerase I inhibitor	∼4	Cys (site-specific)	NSCLC (non-squamous)	Jan 17, 2025
**Emrelis**	Telisotuzumab Vedotin	c-MET	IgG1	Enzyme cleavable	MMAE/auristatin	Microtubule inhibitor	∼3.1	Cys (random)	MET-overexpressing NSCLC	May 14, 2025

**Abbreviations:** DAR: drug-to-antibody ratio; DM1, emtansine; DM4, ravtansine; DXd, deruxtecan; MMAE, monomethyl auristatin E, MMAF, monomethyl auristatin F; AML, acute myeloid leukemia; cHL, classical Hodgkin lymphoma; sALCL, systemic anaplastic large cell lymphoma; PTCL, peripheral T-cell lymphoma; BC, breast cancer; B-ALL, B-cell acute lymphoblastic leukemia; DLBCL, diffuse large B-cell lymphoma; UC, urothelial carcinoma; GC, gastric cancer; NSCLC, non–small-cell lung cancer; mTNBC, metastatic triple-negative breast cancer; HR+/HER2– BC, hormone receptor–positive/HER2-negative breast cancer; MM, multiple myeloma; CC, cervical cancer; OvCa, ovarian cancer.

*1 Reapproved on Sep 1, 2017.

*2 Reapproved on Oct 23, 2025.

*Note*: moxetumomab pasudotox (LUMOXITI) was approved by the FDA on Sep 13, 2018 for hairy cell leukemia, but it was withdrawn from the U.S. market by the sponsor on Nov 18, 2022 due to lack of use, complexity of administration, and availability of other treatment options.

Clinical performance of approved ADCs varies widely. For example, gemtuzumab ozogamicin (GO), the first FDA-approved ADC for relapsed or refractory acute myeloid leukemia (AML),^17^ demonstrated early promise but encountered significant challenges achieving long-term efficacy due to acquired resistance and severe side effects.^18^ Trastuzumab DXd (T-Dxd), first approved in 2019 for HER2-positive breast cancer,^19^ has demonstrated high efficacy and durable response, leading to approvals in 2021 for HER2-positive gastric cancer,^20^ in 2022 for HER2-mutant non-small cell lung cancer (NSCLC),^21^ and most recently for unresectable or metastatic tumors with HER2 IHC 3+ expression. However, despite the linker design for tumor-selective cleavage, systemic Dxd exposure can reach levels comparable to those observed in clinical studies of DX-8951f, the cytotoxic component of Dxd.^22^ Patients frequently experience prolonged and severe nausea and vomiting after T-Dxd administration, which limits tolerability in some patients.

## Mechanisms of resistance to ADCs

Despite the advances and clinical benefits, primary resistance mechanisms that prevent ADCs from being effective from the outset of therapy and acquired resistance after initial sensitivity to ADCs remain significant challenges. Here, we outline the major molecular pathways that contribute to both primary and acquired resistance, including altered antigen expression, increased drug efflux, and impaired apoptotic signaling. As ADCs are evaluated and approved across an increasingly broad range of cancer types and clinical settings, it will be essential to understand and overcome these mechanisms to increase the efficacy and durability of ADC therapy.

### Tumor heterogeneity, low antigen expression, and antigen downregulation

Tumor heterogeneity refers to the natural variation in drug sensitivity and antigen expression among cancer cells within the same tumor. Each tumor’s structure, vasculature, cellular, enzymatic, and antigen composition can all impact the ability of an ADC to effectively penetrate and deliver its payload to tumor cells.^23^ Before treatment, certain cells may express the target antigen at high levels, while others exhibit low or undetectable levels. This translates to ADC efficacy in antigen-high cells, though antigen-low or antigen-negative populations survive, proliferate, and eventually drive tumor progression.^24–26^ Tumor heterogeneity may vary between tumors or over time within a patient, between patients, and between diseases. For example, intra-tumoral HER2 expression heterogeneity is more prevalent in HER2-positive gastric cancer than in HER2-positive breast cancer,^27^ which may affect the efficacy of T-Dxd in this context. During treatment, cancer cells can develop acquired resistance to ADCs by modulating target antigen expression via downregulation or mutation (Figure 1C). This is demonstrated with most ADCs, and examples include T-DM1 with loss of HER2 expression in breast cancer,^28–31^ brentuximab vedotin (BV) with loss of CD30 in anaplastic large cell lymphoma,^32^^,^^33^ and sacituzumab govitecan (SG) with diminished binding affinity in patients with triple negative breast cancer (TNBC) carrying the TROP2^T256R^ missense mutation at relapse.^7^^,^^29^ These examples underscore antigen modulation as a significant challenge to the sustained efficacy of ADC therapies.

### Altered antigen internalization and recycling

Following ADC binding to target antigen, effective therapy requires internalization and trafficking to lysosomes for payload release. ADC-antigen complexes are internalized via (i) clathrin-mediated endocytosis, the primary pathway for ADC uptake, (ii) caveolae-mediated endocytosis, and (iii) macropinocytosis. In T-DM1-resistant gastric cancer cell lines, HER2 receptor internalization was shown to occur preferentially via caveolae-mediated endocytosis, with reduced lysosomal colocalization, limiting payload delivery.^34^

### High expression of drug efflux pumps

Once internalized and cleaved, ADC cytotoxic payload must reach its intracellular target. However, some cancer cells overexpress multi-drug resistance transporters, such as MDR1, which actively pumps cytotoxic payload out of the cell, thus reducing efficacy. Cancer cell overexpression of efflux pumps is associated with resistance to various chemotherapeutics and is a common barrier in ADC therapy. For example, MDR1 expression in AML blasts correlates with ADC resistance and poor patient outcomes with GO and inotuzumab ozogamicin (IO), which has the same MDR1 substrate payload (calicheamicin) as GO.^35–37^ Resistance due to drug efflux pump expression may occur at baseline or be acquired after ADC exposure, such as with MDR1 and GO-resistant AML,^33^ BV-resistant Hodgkin lymphoma,^33^^,^^38^^,^^39^ T-Dxd-resistant breast cancer,^40–42^ and with increased expression of ABCC2 and ABCG2 in T-DXd-resistant gastric cancer.^43^

### Modulation of apoptotic pathways

Primary or acquired resistance can develop if the apoptotic machinery is inherently dysfunctional. For example, defective activity of pro-apoptotic proteins such as BAX and BAK, or constitutive overexpression of anti-apoptotic proteins like BCL-2 and BCL-xL, can impair apoptosis.^44^ In fact, insufficient activation of BAX and BAK and overexpression of BCL-2 and BCL-xL in AML are associated with GO resistance.^45^^,^^46^ Similarly, high BCL-2 expression in non-Hodgkin lymphoma^47^^,^^48^ and downregulation of the pro-apoptotic protein Bim likely contribute to primary and acquired resistance to polatuzumab vedotin.^49^^,^^50^ These examples highlight that both initial response and sustained efficacy may be compromised by the tumor’s inherent or acquired ability to remodel apoptotic pathways under therapeutic pressure.

### Lysosomal sequestration and impaired payload release

Once internalized, complete release of the cytotoxic payload is achieved when ADCs are degraded in the lysosome. However, alterations in the lysosomal environment, such as elevated pH or decreased protease activity, may hinder linker cleavage and prevent payload release. Activation of the PI3K/AKT/mTOR pathway in cancer cells is associated with lysosomal dysfunction, thus contributing to both primary and acquired resistance.^51–53^ For instance, lysosomal alkalinization and reduced proteolytic activity renders T-DM1-resistance in breast cancer cell lines.^54^ Additionally, loss of SLC46A3, a lysosomal membrane transporter of the active metabolite of T-DM1 (Lys-SMCC-DM1), has been linked to resistance and may also preexist in some tumors, contributing to primary resistance.^40^^,^^55^

## Strategies to overcome resistance to ADCs

Multiple ADC engineering and synergistic drug combination strategies are being explored to overcome the various mechanisms of resistance to ADC therapies. With proof of concept demonstrated in key animal studies (Table 2), these strategies aim to enhance ADC delivery and cytotoxicity by optimizing ADC internalization and payload release while addressing resistance mechanisms, as summarized in Figure 1C.

**Table 2.** Preclinical animal models for studying resistance to antibody-drug conjugates (ADCs).

Strategy	Model	ADC/combination	Mechanistic rationale	Key outcome
**Overcoming antigen downregulation, lysosomal dysfunction, and drug efflux**	HER2-positive breast cancer PDX (T-DM1–resistant)^56^	SYD985 (trastuzumab duocarmazine)	Cleavable linker and membrane-permeable duocarmycin payload enable bystander killing and reduce dependence on antigen density, lysosomal processing, and MDR1-mediated efflux	Maintained antitumor activity in HER2-low and T-DM1–resistant models
**Enhancing lysosomal biogenesis via mTORC1 inhibition**	HER2-positive breast cancer xenograft & PDX^57^	T-DM1 + everolimus	mTORC1 inhibition enhances lysosomal biogenesis and trafficking, increasing lysosomal delivery and degradation of T-DM1 and improving intracellular payload release	Restored antitumor activity of T-DM1 in HER2-positive xenograft and PDX models
**Addressing tumor heterogeneity, payload sensitivity variability, and drug efflux**	HER2-positive breast cancer xenograft^58^	MMAE/MMAF dual-payload ADC	Dual-payload design combines membrane-permeable MMAE–mediated bystander killing with MMAF activity that is less susceptible to MDR1-mediated efflux, enabling activity across heterogeneous tumor cell populations	Improved antitumor efficacy and reduced emergence of resistant clones compared with single-payload ADCs
**Targeting HER2 heterogeneity and low antigen expression via biparatopic ADC**	HER2-positive breast cancer xenograft & PDX^59^^,^^60^	ZW49 (zanidatamab zovodotin)	Dual-epitope HER2 engagement is designed to improve internalization and intracellular payload delivery across heterogeneous HER2 expression compared with monospecific HER2 ADCs	Significant antitumor activity in HER2-expressing mouse xenograft models with heterogeneous or low antigen expression
**Overcoming drug-efflux–mediated resistance**	Nectin-4–positive, MDR1-high breast cancer xenograft^61^	Anti–Nectin-4–MMAE ADC + tariquidar	Pharmacologic inhibition of MDR1 by tariquidar increases intracellular retention of MMAE, restoring effective cytotoxic concentrations within tumor cells	Restored antitumor efficacy of the MMAE-based ADC in MDR1-high xenograft models
**Overcoming drug-efflux–mediated resistance**	Hodgkin lymphoma xenograft (BV-resistant)^38^	BV+ cyclosporine A	Cyclosporine A suppresses MDR1-mediated efflux, leading to increased intra-tumoral MMAE accumulation and enhanced cytotoxic activity of BV	Re-sensitization of MDR1-mediated BV-resistant tumors and restoration of antitumor activity in vivo

### Targeting tumor heterogeneity and antigen downregulation

Tumor heterogeneity and downregulation or loss of target antigens are the main causes of resistance. To address these challenges, several strategies have been pursued.

#### Next-generation ADCs targeting a broader range of antigens

Antigen loss and intra-tumoral heterogeneity may be addressed by bi-specific or multi-specific ADCs that target multiple epitopes or antigens. These ADCs increase the likelihood of payload delivery in tumors with heterogeneous antigen expression. Examples include ZW49 (briefly summarized in Table 2) and TQB2102, which are bi-specific HER2-targeted ADCs, each engineered to recognizing two distinct HER2 epitopes, a design intended to improve target engagement and payload release in tumors with heterogeneous HER2 expression. TQB2102 binds HER2 on extracellular domains 2 and 4 (ECD2 and ECD4) and incorporates an enzyme-cleavable linker conjugated to a topoisomerase I inhibitor payload. Phase II clinical evaluation of TQB2102 demonstrates promising antitumor activity with a manageable safety profile in HER2-expressing breast cancer.^62^ These emerging clinical data support the translational potential of bi-specific ADCs as a next-generation strategy to overcome resistance to earlier HER2-directed therapies.

#### ADCs with bystander effect

As mentioned above, T-Dxd demonstrates notable efficacy in HER2-positive gastric cancer despite intra-tumoral heterogeneity of target expression. This is partly due to the bystander effect,^27^^,^^63^ whereby the ADC exerts therapeutic effects beyond the directly targeted cells as cytotoxic payload diffuses into neighboring antigen-negative or low-antigen-expressing cells after being released by antigen-positive cells. Bystander effect relies somewhat on linker properties but more so on payload membrane permeability.^64–66^ Examples include trastuzumab-based ADCs, SYD985 (highlighted in Table 2) and T-Dxd, which exhibit bystander effect due to their respective membrane-permeable DNA binder/alkylator (duocarmazine) and topoisomerase I inhibitor payloads (Dxd).^67^^,^^68^ Dxd, a derivative of camptothecin analog DX-8951f, demonstrated significant antitumor activity in early clinical trials as a standalone agent.^22^^,^^69–71^ The DESTINY-Breast03 trial showed that in patients with HER2-positive metastatic breast cancer, T-Dxd was associated with a lower risk of disease progression or death compared with T-DM1, which has no expected bystander effect.^72^ While the bystander effect can improve ADC efficacy in heterogeneous tumors, excessive diffusion of the cytotoxic drug may harm normal surrounding tissues, highlighting the need for balance between potency and selectivity during ADC design. Newer ADCs with enhanced bystander effect are under development.^73^^,^^74^

#### Modulation of target antigen

Low antigen expression poses a major challenge for ADC efficacy, though expression of some antigens may be modulated pharmacologically to enable ADC binding and internalization. For example, CD33 is highly expressed in undifferentiated myeloid progenitor cells, though expression levels tend to decrease during differentiation.^75–77^ Inhibitors of glycogen synthase kinase 3 alpha and beta (GSK3α/β) can upregulate CD33 expression in AML cells,^78^ and although the exact mechanism is unclear, GSK3α/β may also control cell differentiation.^79^^,^^80^ Therefore, GSK3α/β inhibition may prevent GO resistance in AML by restoring and maintaining CD33 expression within the undifferentiated state of myeloid progenitor cells.

### Improving internalization and payload release

Effective internalization and release of payload are critical for ADC efficacy, and several approaches have been investigated to enhance this process.

#### Optimization of linkers

The balance of linker stability and release efficiency is critical for ADC safety and efficacy. Cleavable, protease-sensitive or pH-sensitive linkers aim to increase the proportion of payload selectively released into cancer cells or the tumor microenvironment. For example, the valine-citrulline linker used in BV is cleaved by cathepsins in lysosomes, enhancing the controlled and targeted release of the cytotoxic payload.^81^ Non-cleavable linkers, such as in T-DM1, require complete lysosomal degradation of the antibody for payload release, thus increasing systemic stability and safety.^82^ Linker optimization to balance stability and efficient release at the tumor site is key to reducing off-target toxicity and enhancing therapeutic efficacy.

#### Lysosomal activation and trafficking

The acidic environment of lysosomes is crucial for linker cleavage and release of many ADC payloads. mTORC1 regulates lysosomal activity, and activation of the PI3K/AKT/mTOR pathway impairs lysosomal function in various cancers.^52^^,^^83^ GSK3 inhibition regulates lysosomal function via mTORC1,^84^^,^^85^ and our previous work demonstrated mTORC1/2 inhibition suppressed p70S6K and AKT phosphorylation, thus enhancing lysosomal activity and GO cytotoxicity.^86–88^ Similarly, mTORC1 inhibition via everolimus enhanced efficacy of T-DM1 in murine tumor models (Table 2). We also demonstrated GSK3α/β inhibitors combined with GO suppress p70S6K phosphorylation and enhance lysosomal function and GO-induced cell death.^78^ These findings suggest combined mTORC1/2 or GSK3α/β inhibitors with ADCs overcomes lysosomal dysfunction, thus facilitating linker cleavage, payload release, and improved efficacy.

Enhanced delivery to lysosomes may also be achieved by inhibiting the neonatal Fc receptor (FcRn), which recycles IgG antibodies and salvages them from lysosomal degradation.^14^ Studies with FcRn inhibitors such as efgartigimod have demonstrated increased IgG degradation in lysosomes, which may enhance payload release, particularly when lysosomal dysfunction limits linker cleavage and ADC degradation.^89^

### Inhibiting drug efflux

Drug efflux transporters confer resistance by actively expelling cytotoxic payloads, and various strategies have been developed to overcome this.

#### Combination therapy with efflux pump inhibitors

Inhibitors of drug efflux pumps can enhance cancer cell retention of cytotoxic payloads. Verapamil, cyclosporine A and tariquidar inhibit MDR1 activity and can increase intracellular payload concentrations when combined with ADCs, thereby improving therapeutic efficacy (see examples in Table 2).^90^^,^^61^ Cyclosporine A, which inhibits MDR1 expression, restored therapeutic effect of BV when used in combination in patients with MDR1-expressing, acquired BV-resistant Hodgkin’s lymphoma.^91^ Although efflux pump inhibitor combinations with cytotoxic drugs have been limited clinically due to off-target effects and systemic toxicities, this example indicates that overcoming the payload resistance mechanism facilitates reversal of ADC resistance.^92^ Furthermore, as ADCs selectively deliver cytotoxic payload to antigen-positive tumor cells, combination with efflux pump inhibitors may allow enhanced efficacy without proportionally increasing toxicity to normal tissues.

#### Payload modification

ADC payloads can be chemically modified to alter their efflux pump susceptibility, increasing intracellular retention and efficacy. For example, MMAE is membrane-permeable and capable of inducing a bystander effect but is more susceptible to MDR-1-mediated efflux, whereas monomethyl auristatin F (MMAF) is membrane-impermeable and generally less affected by efflux (see example in Table 2).^65^ Another strategy is hydrophilic linkers such as PEG_4_Mal, which can lower payload efflux by reducing interaction with MDR1.^93^ Dual-drug ADCs, incorporating two distinct payloads, can also mitigate MDR1-mediated efflux by attaching at least one payload with low MDR1 affinity, such as pyrrolobenzodiazepine.^94^^,^^95^ This strategy enhances intracellular persistence of cytotoxic drugs, leading to improved ADC efficacy in multidrug-resistant cancer cells.

#### Modulation of drug efflux pump expression

Beyond modulation of CD33 expression and cell differentiation, as mentioned above, GSK3α/β signaling also regulates MDR1 expression in some cancers. This was shown with GO-resistant AML cell lines, whereby GSK3α/β inhibition suppressed MDR1 expression and increased calicheamicin intracellular retention.^78^ We also found IKKβ inhibitors suppressed MDR1 mRNA levels in Hodgkin lymphoma cell lines and synergistically enhanced efficacy in combination with BV.^90^ Thus, combinations with antitumor therapies that downregulate drug transporter expression may directly enhance ADC potency and synergistically overcome or delay drug resistance.

### Modulating apoptotic pathways

Resistance to ADCs can occur when cancer cells evade apoptosis, the programmed cell death mechanism cytotoxic payloads attempt to induce. Strategies to overcome apoptosis evasion, include BH3 mimetics and modulation of apoptosis factor expression. BH3 mimetics are small molecules that mimic activity of BH3-only proteins, pro-apoptotic factors that bind anti-apoptotic proteins (e.g., BCL-2, BCL-xL, MCL-1), displacing and liberating other pro-apoptotic factors (e.g. BAX, BAK), leading to apoptosis. For example, the BCL-2 inhibitor venetoclax amplifies apoptosis and enhances cytotoxicity in AML and ALL cell lines when combined with ADCs, including GO and IO, and these combinations are currently under evaluation in clinical trials.^45^^,^^96^^,^^97^ Similarly, the BCL-2/BCL-xL inhibitor navitoclax has shown efficacy in preclinical studies, demonstrating potential to overcome resistance to BV and T-DM1 via similar mechanisms.^98^^,^^99^

Overexpression of anti-apoptotic proteins is a common mechanism of ADC resistance, yet studies specifically targeting expression of anti-apoptotic factors remain limited. GSK3α/β inhibitors can downregulate expression of BCL-2 family proteins in various cancer models,^100–103^ and we previously demonstrated combined GSK3α/β inhibitors and GO enhanced apoptotic cell death.^78^ These examples highlight the potential of targeting anti-apoptotic pathways to improve ADC efficacy in resistant cancers.

### Combination with other anticancer modalities to overcome resistance

Combination therapy that simultaneously targets multiple resistance pathways is a promising strategy for overcoming ADC resistance and increasing therapeutic efficacy. The first ADC combination was approved in 2017,^104^ which was GO+daunorubicin and cytarabine (ADC+chemo) in patients with CD33-positive AML. Several ADC+chemo FDA approvals followed, highlighting early thinking with ADC combinations and standard-of-care therapies.

#### Combination with chemotherapy

As discussed, chemotherapy can be combined with ADCs to enhance therapeutic efficacy through complementary mechanisms. Many ADC+chemotherapy regimens have been evaluated, as recently reviewed.^105^ While these combinations sometimes increase adverse events, many have demonstrated improved efficacy with acceptable tolerability.^106^ For example, combining BV with the AVD chemotherapy regimen (adriamycin, vinblastine, dacarbazine) demonstrated promising results in patients with Hodgkin lymphoma and was ultimately FDA approved.^107^^,^^108^ Additionally, chemotherapy can support the activity of ADCs by reducing tumor burden or modulating resistance pathways, contributing to overall therapeutic efficacy.

Beyond chemotherapy, many ADC combinations with other agents have been evaluated clinically. Late-stage trials with ADC combinations represent therapies nearing approval, and 20 of these studies have been initiated in the past 5 years (19 phase 3 and 1 phase 4). Table 3 summarizes these studies and lists general trends of combination rationale. Though chemotherapy is represented in 4 of these trials, combinations with immunotherapy represent the majority (12), and targeted therapies fall in between (6). Notably, 3 of 8 trials that include targeted therapies also include immunotherapy (2) or chemotherapy (1).

**Table 3.** Late-phase clinical trials evaluating antibody-drug conjugates (ADCs) in combination regimens with other agents.

Trial number	Start date	ADC(s) investigated	Combination partner(s)/therapy type	Combination category	Indication
**NCT07162259**	10/1/2025	SG, T-DXd	Each other (sequential)	ADC + ADC	Breast cancer
**NCT07239271**	11/16/2025	T-DM1, T-DXd, SHR-A1811	Chemotherapy (various)	ADC + chemotherapy	Breast cancer
**NCT06520345**	7/26/2024	177Lu-TLX591 (rADC)	Enzalutamide, Abiraterone, Docetaxel	rADC + chemo/targeted	Prostate cancer
**NCT05445778**	12/27/2022	Mirvetuximab Soravtansine	Bevacizumab	ADC + targeted therapy	Ovarian cancer
**NCT05904964**	7/1/2023	Disitamab Vedotin	Endocrine Therapy	ADC + targeted therapy	Breast cancer
**NCT06868654**	7/28/2021	Belamaf	Bortezomib, Dexamethasone	ADC + targeted therapy	Multiple myeloma
**NCT06868667**	7/28/2021	Belemaf	Bortezomib, Dexamethasone	ADC + targeted therapy	Multiple myeloma
**NCT06956170**	2/10/2022	Belemaf	Pomalidomide, Dexamethasone	ADC + targeted therapy	Multiple myeloma
**NCT05687266**	12/29/2022	Dato-DXd	Durvalumab, Carboplatin	ADC + immuno + chemo	Non-small Cell Lung Cancer
**NCT06112379**	11/14/2023	Dato-DXd	Durvalumab, Chemotherapy	ADC + immuno + chemo	Breast cancer
**NCT04887870**	6/29/2021	EV	Sitravatinib, Nivolumab, Pembrolizumab	ADC + immuno + targeted	Solid malignancies
**NCT07216703**	12/12/2025	Sac-TMT	Pembrolizumab, Bevacizumab	ADC + immuno + targeted	Cervical cancer
**NCT05609968**	2/6/2023	SG	Pembrolizumab	ADC + immunotherapy	Non-small Cell Lung Cancer
**NCT05629585**	11/28/2022	Dato-DXd	Durvalumab	ADC + immunotherapy	Breast cancer
**NCT06103864**	11/23/2023	Dato-DXd	Durvalumab, Pembrolizumab	ADC + immunotherapy	Breast cancer
**NCT06524544**	12/2/2025	SG	Pembrolizumab	ADC + immunotherapy	Urothelial carcinoma
**NCT06758401**	7/23/2025	Sigvotatug Vedotin	Pembrolizumab	ADC + immunotherapy	Non-small Cell Lung Cancer
**NCT06841354**	3/16/2025	Sac-TMT	Pembrolizumab	ADC + Immunotherapy	Breast cancer
**NCT06952504**	5/22/2025	Sac-TMT	Pembrolizumab	ADC + Immunotherapy	Endometrial cancer
**NCT06989112**	3/27/2025	T-DXd	Rilvegostomig, Pembrolizumab	ADC + Immunotherapy	Endometrial cancer

Clinicaltrials.gov was searched on December 18, 2025 using (“Antibody-drug Conjugate” or “Antibody-Drug Conjugate” or “Antibody-drug Conjugates”) AND (“combination” OR “Combination”) from January 1, 2021 to December 18, 2025. A total of 179 studies were identified, and when filtering on Phase 3 or Phase 4 (only NCT07162259) and reviewing study details to confirm ADC combinations, these 20 studies remained. NCT07162259 and NCT07239271 are not yet recruiting, NCT04887870 was completed, and all others are active or active-not recruiting.

**Abbreviations:** SG, sacituzumab govitecan; T-DM1, trastuzumab emtansine; T-DXd, trastuzumab deruxtecan; SHR-A1811, trastuzumab rezetecan; Sac-TMT, sacituzumab tirumotecan; rADC, radioantibody-drug conjugate; Belamaf, belantamab mafodotin; Dato-DXd, datopotamab deruxtecan; EV, enfortumab vedotin.

#### Combination with immunotherapy

ADC combination approaches have been reviewed recently,^105^^,^^106^ though it is worth further highlighting these, particularly ADC combinations with immune checkpoint inhibitors (ICIs) which offer a unique synergistic strategy to enhance antitumor efficacy.^109^ Immune checkpoint inhibitors such as nivolumab or pembrolizumab, can reinvigorate exhausted T cells, complementing direct cytotoxic effects of ADCs. Several combinations have already gained regulatory approval. For instance, the combination of enfortumab vedotin (EV; anti-nectin-4 ADC) and pembrolizumab has been approved for treatment of previously untreated locally advanced or metastatic urothelial carcinoma, based on significant improvements in overall response rates and survival outcomes.^110^ Similarly, in relapsed or refractory classical Hodgkin lymphoma, combination of BV and nivolumab demonstrated promising efficacy, although this combination has not yet received formal FDA approval.^111^

Beyond these, several other ADC+ICI combinations are under clinical investigation.^112^^,^^113^ Early-phase trials of T-Dxd combined with ICIs have shown promising results, particularly in patients with HER2+ and even HER2-low tumors.^114^ In TNBC, early-phase trials of SG (anti-TROP2 ADC) plus pembrolizumab have also shown encouraging antitumor activity.^115^ ICIs may also modulate the tumor microenvironment to favor immune infiltration and reduce immunosuppressive signals, thereby potentiating ADC activity.^116^^,^^117^ This combination approach holds promise for overcoming resistance and enhancing therapeutic responses in certain tumor types.

#### Combination with radiation therapy

Combining ADCs with radiation therapy offers a promising strategy to enhance cancer treatment efficacy through multiple synergistic mechanisms. Radiation can directly kill tumor cells and also modulate the tumor microenvironment to increase antigen expression and promote immune activation.^118^^,^^119^ One late phase trial highlighted in Table 3 (NCT06520345) is evaluating 177Lu-TLX591, a radio antibody-drug conjugate (rADC) for direct targeting of radiation to PSMA-expressing prostate cancer. Other experimental ADC payloads possess radiosensitizing properties, further amplifying radiation’s cytotoxic effects when combined.^120^^,^^121^ This dual mechanism (radiation-induced sensitization of tumor cells and radiosensitizing effects of ADC payloads) can enhance DNA damage and promote tumor regression. Early-stage clinical trials are currently investigating the combination of ADCs with radiation therapy, showing promising potential for improving treatment outcomes in various cancer types.^122^^,^^123^

### Emerging biomarkers of ADC response and resistance

Refinements in antigen quantification—such as HER2-low and HER2-ultralow classifications—and AI-assisted digital pathology may improve the assessment of antigen levels and spatial heterogeneity.^124^ Liquid biopsy approaches can detect *ERBB2* alterations and emerging resistance mutations, enabling real-time monitoring of tumor evolution.^125^ Payload-specific biomarkers such as SLFN11 may predict sensitivity to topoisomerase-I inhibitor ADCs.^126^ Fcγ receptor polymorphisms and FcRn variability, known to influence IgG1 pharmacokinetics, remain exploratory for ADCs and their impact on ADC clearance has not yet been defined.^127^ Although none are established for routine clinical use, these biomarkers may help refine ADC selection and anticipate emerging resistance.

Finally, as listed in Table 3, NCT07162259 is unique as the only phase 4 trial and also the only trial evaluating combined ADCs (sequential T-Dxd and SG in patients with CDK4/6 inhibitor refractory HR+/HER2- breast cancer). While not technically a combination regimen since these agents are given sequentially, it is included as an example of current thinking for use of biomarkers (HER2 expression) and multiple ADCs within the same patient to overcome ADC resistance.

## Emerging ADC technologies

**Figure 2.**
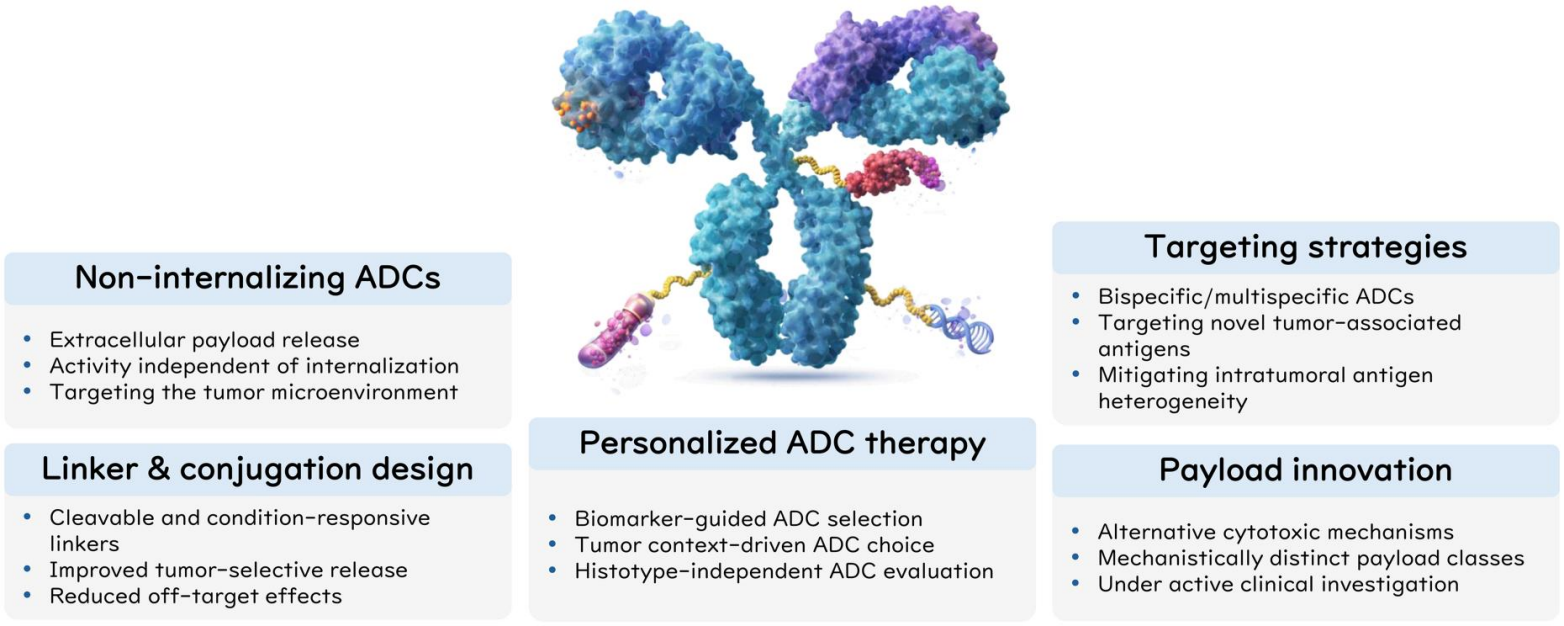
Emerging ADC technologies and evolving therapeutic strategies to address resistance. This figure illustrates emerging ADC technologies and strategies to overcome resistance, including innovations in payloads, linker, and conjugation design, and targeting approaches to address impaired internalization, altered trafficking, and intra-tumoral antigen heterogeneity. In addition, personalized ADC therapy is highlighted through biomarker-guided patient selection and histotype-independent evaluation, exemplified by the DESTINY-PanTumor02 trial of T-Dxd. The schematic illustration was created with the assistance of ChatGPT.

Recent advancements in ADCs focus on improving efficacy, reducing toxicity, and overcoming resistance. To achieve these goals, researchers are exploring novel payloads and linkers, bi-specific and multi-specific antibodies, and new antigen targets. Additionally, personalized approaches aim to tailor ADC therapy to individual patients, enhancing treatment outcomes. To provide an integrated overview of emerging approaches to overcome resistance to antibody–drug conjugates, key technological innovations and evolving therapeutic strategies are summarized in Figure 2 and Table 4.

**Table 4.** Emerging antibody-drug conjugate (ADC) technologies and strategies under clinical evaluation to overcome resistance.

Technology	Strategy/concept	Examples	Key relevance to resistance
**Non-internalizing ADCs**	Extracellular payload release independent of antigen internalization	First-in-class ADC (TGW101) targeting tumor-associated glycoprotein 72^128^ (Phase I) First-in-class ADC (OMTX705) targeting fibroblast activating protein^129^ (Phase I)	Overcome resistance associated with impaired antigen internalization or trafficking
**Linker & conjugation design**	Cleavable and condition-responsive linker systems	Disitamab vedotin (RC48-ADC) with a protease-cleavable linker^130^ (Phase III)	Improve tumor-selective release, reduce off-target effects
**Payload innovation**	Cytotoxic mechanisms beyond conventional strategies	Amanitin-based ADC (HDP-101) inhibits RNA polymerase II^131^ (Phase II) Vobramitamab duocarmazine (MGC018), a duocarmycin derivative that is a potent DNA alkylating agent^132^ (Phase II)	Circumvent resistance driven by payload insensitivity, altered cell death signaling, and drug efflux mechanisms
**Targeting strategies**	Multivalent or target-level antigen engagement	Bi-specific ADC (TQB2102) targeting two different Her2 epitopes (ECD2 and ECD4) (Phase III) Vobramitamab duocarmazine (MGC018) targeting tumor-specific B7-H3 (CD276)^132^ (Phase II) Sigvotatug vedotin (SGN-B6A) targeting integrin β6^133^ (Phase III)	Overcomes resistance associated with limited target specificity and intra-tumoral heterogeneity by exploiting tumor-selective antigen expression, enabling the use of highly potent cytotoxic payloads
**Personalized ADC therapy**	Biomarker-driven selection of patients and ADC therapies	T-Dxd (DESTINY-PanTumor02)^134^ (Phase II) Dato-Dxd (TROPION-PanTumor03)^135^ (Phase II) Various (ADC-Match)^63^ (Phase II)	Mitigates resistance driven by tumor heterogeneity and histotype-specific limitations by integrating biomarker-guided patient selection with histotype-independent ADC evaluation

## Future directions and conclusion

Although this review did not frame the resistance mechanisms within a pharmacokinetic context, many resistance mechanisms are fundamentally pharmacokinetic in nature. Future research will focus on enhancing and fine-tuning linker stability, developing novel targeting strategies, and optimizing payloads to further improve efficacy and reduce toxicity. Advances in multi-omics and artificial intelligence-driven drug discovery will aid in the identification of novel tumor-associated antigens, enabling more precise and personalized ADC therapies. Additionally, combination strategies with ICIs, kinase inhibitors, or other targeted therapies may help overcome resistance and extend the therapeutic benefits of ADCs.

In conclusion, ADCs have significantly transformed cancer treatment by providing targeted and effective therapy with overall reduced systemic toxicity. However, opportunities remain for improving the therapeutic index of ADCs, and resistance mechanisms remain a major hurdle, thus necessitating continuous innovation. By leveraging new technologies, biomarker-driven approaches, and novel treatment strategies, the next generation of ADCs holds promise for further improving patient outcomes and expanding their clinical applications across diverse cancer types.

## Data Availability

No new data were generated or analyzed for this review article.
